# Modular horizontal network within mouse primary visual cortex

**DOI:** 10.3389/fnana.2024.1364675

**Published:** 2024-04-08

**Authors:** Andreas Burkhalter, Weiqing Ji, Andrew M. Meier, Rinaldo D. D’Souza

**Affiliations:** ^1^Department of Neuroscience, Washington University School of Medicine, St. Louis, MO, United States; ^2^Department of Speech, Language and Hearing Sciences, College of Engineering, Boston University, Boston, MA, United States

**Keywords:** mouse, primary visual cortex, horizontal connections, layer 1, clustered inputs, clustered apical dendrites, M2 muscarinic acetylcholine receptor, patches

## Abstract

Interactions between feedback connections from higher cortical areas and local horizontal connections within primary visual cortex (V1) were shown to play a role in contextual processing in different behavioral states. Layer 1 (L1) is an important part of the underlying network. This cell-sparse layer is a target of feedback and local inputs, and nexus for contacts onto apical dendrites of projection neurons in the layers below. Importantly, L1 is a site for coupling inputs from the outside world with internal information. To determine whether all of these circuit elements overlap in L1, we labeled the horizontal network within mouse V1 with anterograde and retrograde viral tracers. We found two types of local horizontal connections: short ones that were tangentially limited to the representation of the point image, and long ones which reached beyond the receptive field center, deep into its surround. The long connections were patchy and terminated preferentially in M2 muscarinic acetylcholine receptor-negative (M2-) interpatches. Anterogradely labeled inputs overlapped in M2-interpatches with apical dendrites of retrogradely labeled L2/3 and L5 cells, forming module-selective loops between topographically distant locations. Previous work showed that L1 of M2-interpatches receive inputs from the lateral posterior thalamic nucleus (LP) and from a feedback network from areas of the medial dorsal stream, including the secondary motor cortex. Together, these findings suggest that interactions in M2-interpatches play a role in processing visual inputs produced by object-and self-motion.

## Introduction

Navigating in the environment crowded with landmarks and moving objects, depends on neural networks that are sensitive to optic flow, enable segmentation of figures from the background and are capable to distinguish self-generated visual motion from external object motion (Keller and Mrsic-Flogel, 2018; Saleem, 2020). Evidence from behavioral, electrophysiological and calcium imaging studies in mice suggests that all of these functions depend on interactions between thalamocortical, local horizontal connections within primary visual cortex (V1) and feedback pathways from higher visual areas (Self et al., 2014; Schnabel et al., 2018; Keller et al., 2020; Kirchberger et al., 2021; Luongo et al., 2022; Miura and Scanziani, 2022). Each of these interactions are contextual (Kahn and Hofer, 2018), which in their most basic form cause modulations of responses in the classical receptive field (RF) by inputs from the RF surround (Angelucci et al., 2017). In non-rodent visual cortex such interactions are often conveyed by clustered horizontal intra-areal and inter-areal feedback inputs from retinotopically distant locations, that are tuned to matching stimulus features (Gilbert and Wiesel, 1989; Bosking et al., 1997; Federer et al., 2021; Sui et al., 2021). Interactions between horizontal and feedback networks have been shown in monkey V1 to play a role in grouping of line segments with similar orientations into object contours (Liang et al., 2017).

Unlike in primates cats and tree shrews, rats and mice lack orientation columns (Ohki et al., 2005; Ohki and Reid, 2007). Despite this uniform, seemingly non-columnar organization, the distribution of short intrinsic and long horizontal connections in mouse V1 is non-random (Burkhalter and Charles, 1990; Rumberger et al., 2001; Ko et al., 2013; Cossell et al., 2015; Han et al., 2018), and neurons show orientation-dependent surround suppression (Self et al., 2014). In addition, there is a striking clustering of responses to the direction, speed and coherence of stimulus motion in layer 2/3 of mouse V1 (Ji et al., 2015). To make spatially clustered responses to random dot kinematograms (Marques et al., 2018; Sit and Goard, 2020) useful for signaling of the direction of optic flow (Prince and Born, 2009), local motion cues need to be integrated through networks across large regions of the visuotopic map. This feature is represented in horizontal and feedback networks, which were shown to carry information about motion contrast for the detection of boundaries (Self et al., 2014), caused by the speed of object-and self-motion.

While visual features are non-randomly distributed in L2/3 neurons of mouse V1 (Ji et al., 2015; Kondo et al., 2016; Ringach et al., 2016; Maruoka et al., 2017), the clustering is equally striking in the distribution of dendritic and axonal projections to L1 (D’Souza et al., 2019; Meier et al., 2021). Axons from the dorsal lateral geniculate nucleus (dLGN) and the higher order lateral posterior thalamic nucleus (LP) terminate in L1, where they cluster in M2 muscarinic acetylcholine receptor-rich M2+ patches and M2− interpatches, respectively (D’Souza et al., 2019). In addition, M2+ patches and M2− interpatches are targeted by distinct feedback pathways from higher cortical areas (D’Souza et al., 2021; Burkhalter et al., 2023), indicating that L1 is an important site for coupling bottom-up and top-down inputs onto apical dendrites of V1 pyramidal cells (Larkum et al., 2007; Larkum, 2013; Fisek et al., 2023). It is not known, in any species, how horizontal connections tie into these patchy, L1 targeting networks. To determine this organization we have traced the horizontal connections within mouse V1. We found that the network selectively targets M2− interpatches. Previous studies have shown that feedback inputs from the LP and higher cortical areas (D’Souza et al., 2019; Harris et al., 2019) target M2− interpatches, suggesting that all of these networks converge at these sites. LP inputs provide visual and non-visual signals about the direction of motion (Roth et al., 2016; Miura and Scanziani, 2022), suggesting that the combination of these inputs play a role in the discrimination of self-generated and externally generated visual motion.

## Materials and methods

### Mice

We performed experiments using 5–10 week-old female and male C57BL/6 J (JAX:000664), Ai9 (B6,Cg-Gt[ROSA]26Sor^tm9(CAG-tdTomato)Hze^/J) (JAX:007090), and Pvalb-Cre (B6, 129Ps-Pvalb^tm1(cre)Arbr^/J) (JAX:017320) crossed with Ai9 reporter mice. C57BL/6 J mice were used for anterograde tracing of horizontal connections. Ai9 mice were used for retrograde labeling of cell bodies and dendrites with cre-dependent AAV. Crosses of Pvalb-cre with Ai9 mice were used to identify layers in coronal and tangential sections of V1. All experimental procedures were performed in accordance with the National Institutes of Health guidelines and under the approval of the Washington University Institutional Animal Care and Use Committee.

### Anterograde and retrograde AAV tracing

We labeled axons, cell bodies and dendrites of horizontally-projecting neurons within V1 by tracing with anterograde AAV2/1-hSyn-tdTomato.WPRE.bGHe (Allen Institute) or retrograde AAV2.Retro-CAG.Cre (University of North Carolina, Vector Core), respectively. dLGN inputs to V1 were labeled by AAV2/1-hSyn-EGFP.WPRE.bGHe (University of Pennsylvania, Vector Core) and were used as proxy of M2+ patches (Ji et al., 2015; D’Souza et al., 2019).

### Injections and immunostaining

For tracing connections, mice were anesthetized by intraperitoneal injections of a mixture of ketamine (86 mg/kg) and xylazine (13 mg/kg). Buprenorphine-SR (0.1 mg/kg, subcutaneous) was injected prior to surgery for analgesia. Mice were head-fixed on a stereotactic apparatus. Body temperature was monitored and maintained at 37°C. Craniotomies were made over the injection targets, using a dental drill. Viral injections into V1 and dLGN were delivered via glass micropipettes (tip diameter 20 μm) attached to a Nanoject II pump. V1 injections were made at 1.2 mm in front of the anterior margin of the transverse sinus, 2.8 mm lateral to the midline. Two injections, 46 nL each, were made at 0.3 mm and 0.5 mm below the pial surface. Pipettes were kept in place for 5 min after each injection to allow for diffusion of the tracer. dLGN injections were made 2.35 mm posterior of bregma, 2.15 mm lateral of midline and 2.55 mm below the pial surface. The scalp was stapled and secured with wound clips. Three weeks later, mice were overdosed (ketamine/xylazine 172/26 mg/kg) and perfused with 1% paraformaldehyde (PFA), the cortex was flatmounted. For flatmounting the cortex was separated from the brain and unfolded along the prefrontal, medial and occipital wall, using microsurgical knives (Premier Edge, PE3720, Oasis, CA). The tissue was postfixed in 4% PFA overnight, cryoprotected in 30% sucrose, and cut on a freezing microtome at 40 μm in the tangential plane. The plane of section was perfectly aligned to the pial surface, so that first section through the hemisphere contained only L1.

For immunostaining sections were immersed in blocking solution (0.1% TritonX-100, 10% normal goat serum, PBS) and incubated with a rat anti-M2 muscarinic acetylcholine receptor antibody (M2, 1:500, MAB367, Millipore). M2 was visualized with Alexa-647-tagged goat anti-rat secondary antibody (1:500, A21247, Invitrogen). Sections were wet-mounted on slides and images were acquired under a fluorescence microscope at 2–40X magnification, which were used to delineate M2+ patch borders and to identify labeled cell bodies axons and dendrites.

Layers were identified by the number in a series of consecutive 40 μm tangential sections (Supplementary Figure S2). The approach made use of the lamina-specific distribution of parvalbumin (PV) and the transcription factor, Ctip2 (Arlotta et al., 2005; Palicz et al., 2024). Immunostaining for Ctip2 was performed in tangential sections of Pvalb-Cre mice crossed with Ai9 reporter mice reacted with a rat-anti-Ctip2 antibody (1:500; abcam Cat # ab18465), and visualized with an Alexa-647 tagged goat-anti-rat secondary IgG (1:500, A21247, Invitrogen).

### Delineation of M2+ patches and M2− interpatches

To determine M2+ patches, images of M2 immunostaining or geniculocortical projections were first spatially normalized by dividing the intensity of each pixel by the average intensity from a circle with 100 μm radius surrounding it (D’Souza et al., 2019; Meier et al., 2021). Images were then blurred with a circular averaging filter of 30 μm radius. After that the image was divided into 6 quantiles based on the resulting pixel intensities, with the top 2 quantiles considered to be M2+ patches and the bottom 2 designated M2− interpatches. Automated determination of quantile boundaries was determined with custom MATLAB scripts. To map M2+ patch and M2− interpatch domains onto L2-6, serial tangential sections through V1 were aligned. To correct for tissue distortion due to sectioning and mounting, blood vessels were used as landmarks. Warping was performed using a projective transformation via the MATLAB ‘fitgeotrans’ function. Labeled axonal projections, cell bodies and apical dendrites were assigned to M2+ patches or M2− interpatches depending on which quantile they aligned with.

### Quantification of axonal projection strength

To determine the strength of anterogradely labeled horizontal axonal projections in M2 intensity quantiles, M2 expression images were high-pass filtered and blurred. M2 images were then divided into 6 intensity quantiles. The average optical density of axons in ROIs (550 μm x 400 μm, 10X, 3 per section at different locations where the projections density was strong enough for quantification) within each quantile were then found. The mean and standard error of intensity within each quantile was computed across multiple ROIs per section and subjects and plotted as smooth curves. The Kolmogorov–Smirnov (KS) test was used to compare intensity distributions in M2+ patches and M2− interpatches.

### Quantification of dendrites and cell bodies in M2+ patches and M2− interpatches

The patch/interpatch pattern in L1 was visualized by M2 immunostaining. Fluorescence images were high-pass filtered and blurred, labeling was divided into six intensity quantiles and the top 2 quantiles were designated as M2+ patches while the bottom two 2 were denoted at M2− interpatches. Retrogradely labeled cells were identified in ROIs (550 μm x 400 μm, 10X, 3 per section) within each quantile and plotted manually, using the multi point selection tools of ImageJ (NIH). Retrogradely labeled apical dendrites of V1-projecting cells were identified using morphological features such as thickness, tapering and spine-bearing (Karimi et al., 2019). The density of dendrites was quantified and statistically compared as described for the density of axonal projection strength.

## Results

### Modular specificity of axonal projections within V1

**Figure 1:**
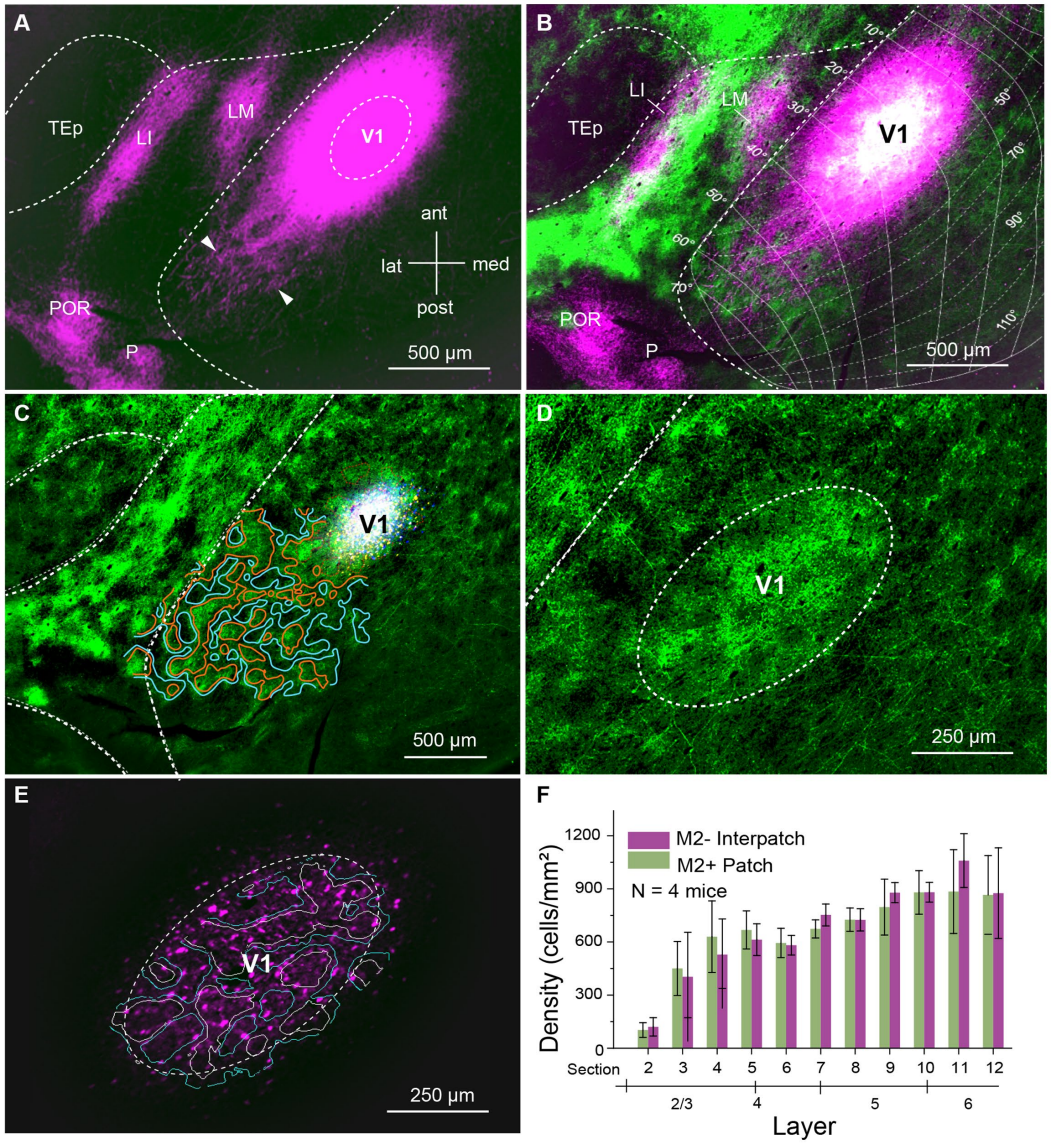
Tangential section through visual cortex of C57BL/6 J mouse. **(A)** Anterogradely labeled horizontal connections in L1 of primary visual cortex (V1) produced by injection of AAV2/1-hSyn-tdT.WPRE.bGHe into V1. The size of the injection appears larger than the effective zone of uptake, due to overexposure of the image, required for revealing the local projections (magenta). Bundles of horizontal fibers exit the intensely fluorescent core of the injection. At increasing tangential distance from the injection center bundles of axons can be resolved, which further down the path form terminal clusters (representative examples indicated by arrow heads) within V1. Projections to cortex surrounding V1, terminate in the higher visual areas, LM (lateromedial), LI (laterointermediate), P (posterior) and POR (postrhinal). **(B)** Spatial alignment of horizontal connections (magenta) with AAV2/1-hSyn-EGFP.WPRE.bGHe labeled inputs (green) from the dorsal lateral geniculate nucleus (dLGN), known to terminate in M2+ patches (Ji et al., 2015; D’Souza et al., 2019). The white region at the center of the injection shows the distribution of dLGN input to M2+ patches. Despite the large injection, which includes M2+ patches and M2− interpatches, the horizontal connections in L1 are preferentially clustered in M2− interpatches. The connections are anisotropic: longer in the axis of elevation and shorter along azimuth, covering about 90° × 50° of visual space. The visuotopic map was adapted from Marshel et al. (2011). **(C)** Center of AAV injection (white). Patchy pattern of dLGN input (green, surrounded by red lines) to L1. Domains surrounded by blue lines represent M2− interpatches. **(D)** Patchy pattern of dLGN input to L1 of V1. **(E)** Labeled cell bodies (magenta) in the zone of effective tracer uptake at the center of the injection. White borders outline M2+ patches. Blue borders surround M2− interpatches. **(F)** Density (cells/mm^2^) of AAV expressing cell bodies in L2-6 in the effective zone of AAV uptake. The graph shows that cells are distributed equally across M2+ patches (green) and M2− interpatches (magenta), which rules out that the selectivity of horizontal connections for M2− interpatches is due to preferential labeling of neurons in interpatches. Error bars represent the standard error of the mean (SEM).

We labeled axonal connections within V1 by anterograde tracing with AAVs in C57BL/6 J mice. Initially, we aimed at confining AAV injections to 80–100 μm-wide columns across L1-6 aligned with M2+ patches or M2− interpatches in L1. M2+ patches were labeled either with an antibody against M2 muscarinic acetylcholine receptor or by anterograde tracing of overlapping geniculocortical inputs to L1 (Ji et al., 2015; D’Souza et al., 2019). We found that small injections (6 mice, 3 injections each) of 10–20 nL administered at two depths of the presumed central part of the binocular visual field (*N* = 4, 20°–40° elevation, 30°–50° azimuth) labeled a small number of cell bodies in L2–6, including their axonal projections. Although individual axons could be traced in the tangential plane over hundreds of microns within V1 and surrounding higher visual areas, the labeling was typically too sparse to quantify the projection density in M2+ patches and M2− interpatches. This result was independent of the retinotopic location of the injection site. We then made larger injections (46 nL) at 300 and 500 μm below the pial surface. This approach produced 200–400 μm-wide injection sites, which contained 200–2,800 (*N* = 4 mice) brightly labeled neurons in L2-6, which were uniformly distributed across M2+ patch and M2− interpatch domains (Figures 1A–E). The ratio of labeled M2+ patch/M2− interpatch neurons varied between 0.53–0.75 (*N* = 4 mice) favoring M2− interpatches, over 1.5–1.7, preferring M2+ patches. Across 4 mice the ratio averaged 0.9–1.24 (Figure 1F). Although we were able to identify labeled cell bodies at the injection core, the intense fluorescence, the freely crossing of fibers and high bouton density made it challenging to distinguish between axonal projections to M2+ patches and M2− interpatches (Figure 1A). However, in the halo surrounding the core, 400–500 μm from the center of the injection, we were able to resolve isolated axon bundles, which at increasingly longer tangential distances formed distinct terminal clusters in L1 (Figure 1A; Supplementary Figure S1). Such clusters were most prominent along the vertical axis (elevation) of the visuotopic map. Along the perpendicular axis (azimuth), projections were shorter and clustering of axons was only notable as a scalloped rim around the injection core (Figure 1B). While the clustered distribution of projections after large AAV injections was notable in the non-columnar mouse (Ohki and Reid, 2007), it was unexpected to find preferential clustering in M2− interpatches (Figure 1B; Supplementary Figure S1), the domains that lacked geniculocortical input and M2 immunostaining (Ji et al., 2015; D’Souza et al., 2019). In L2-6 horizontal axons where wide-spread, sparser than in L1 and notably non-clustered (Supplementary Figure S3).

**Figure 2:**
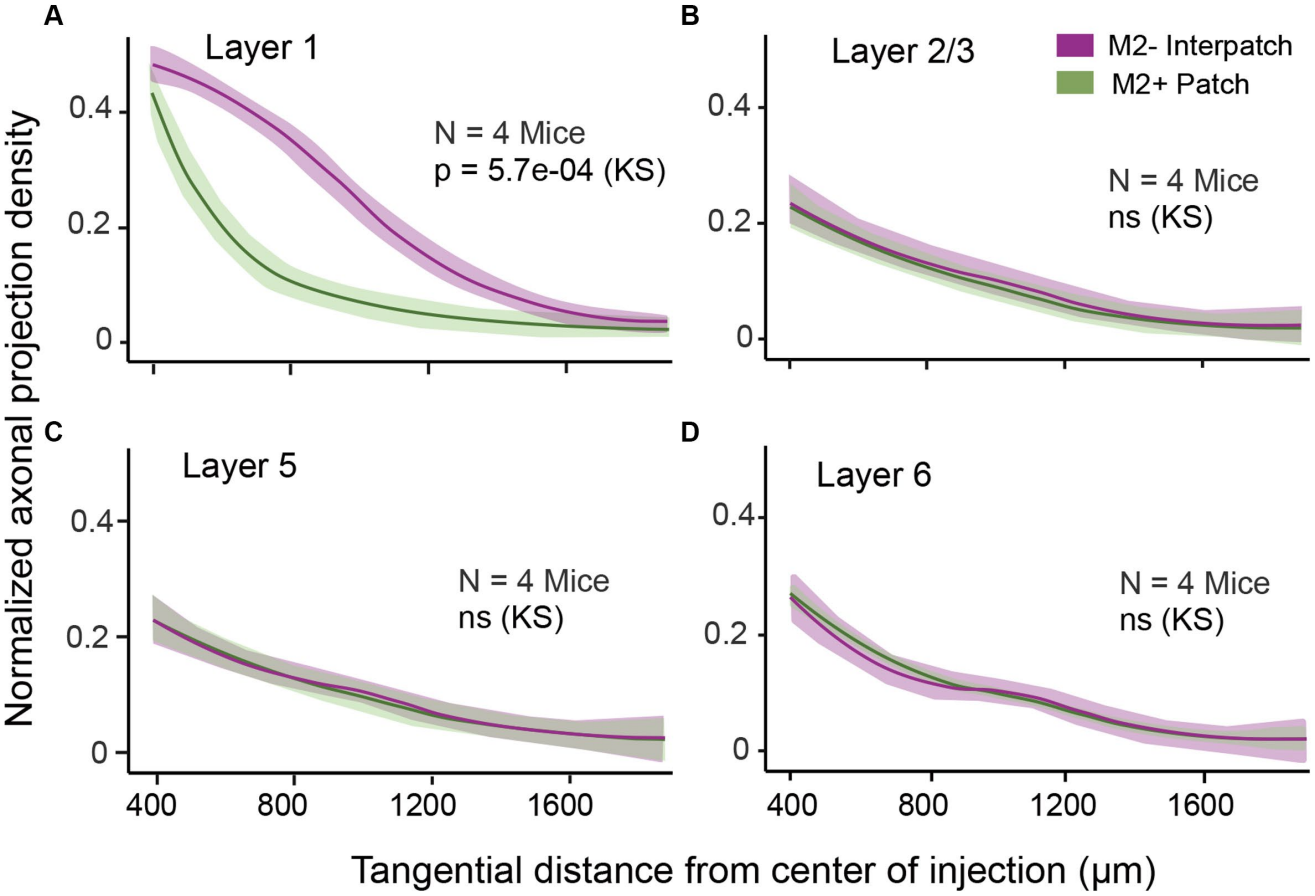
Normalized density of axonal projections as a function of tangential distance from the center of the AAV injection in different layers of V1. The central ring with 250 μm radius is discounted due to intense fluorescence at the core of the injection. **(A)** In L1 the projections are significantly denser in M2− interpatches (magenta) than in M2+ patches (green). **(B–D)** Projections in L2/3 **(B)**, L5 **(C)**, and L6 **(D)** are uniformly distributed across M2+ patches and M2– interpatches. Shading: SEM. KS, Kolmogorov–Smirnov test.

To determine the density of local axonal projections in M2+ patches and M2− interpatches we acquired images of terminal branches and boutons at 10–20× magnification. We then high pass filtered and blurred the ROIs and divided the fluorescence into 6 intensity quantiles. The top 2 quantiles were considered M2+ patches, and the bottom 2 quantiles were designated as M2− interpatches. We then excluded the central 400 μm-wide core of the injection site for analysis of axon density, averaged intensities across 3 ROIs/section/mouse and used Kolmogorov–Smirnov (KS) statistics to test for differences of projections in M2+ patches and M2− interpatches. We found that in L1 projections, contained in sections 1 and 2, to M2− interpatches were significantly (*p* = 5.7e-04; *N* = 12) denser than to M2+ patches (Figure 2A). At the perimeter of the central exclusion zone, the so called halo (Supplementary Figure S1), the projection density in M2+ patches and M2− interpatches was similar. But farther away as clustering in L1 increased (Figure 1B; Supplementary Figure S1), the difference between M2+ patches and M2− interpatches became larger, but finally tailed off when the local projections reached maximal length and became too sparse for quantification. In L2-6 M2+ patch and M2− interpatch domains were assigned by aligning sections using blood vessels as landmarks, and superimposing the patch/interpatch contours of L1. The tangential distribution of local projections in L2-6 were determined in sections 3–16. Because layers are difficult to identify in tangential sections by cytoarchitecture, we used PV to determine the bottom of L4, and Ctip2 to identify the infragranular L5 and 6. Supplementary Figure S2 shows that L2-4 are contained in sections 3–7, L5 in sections 8–12 and L6 in sections 13–16. By applying this laminar mapping scheme we found that local projections in L2-6 are weaker than in L1, and most notably are distributed randomly (Figures 2B–D; Supplementary Figure S3).

The long-range horizontal connections in L1 were strikingly anisotropic. The axis of elongation was roughly parallel to the lateral border of V1 and mapped approximately 90° of elevation in the visuotopic map (Figure 1B). The short axis ran orthogonally, roughly parallel to the horizontal meridian and represented about 60° of azimuth (Figure 1B). Because the extent of labeled connections likely depends on the size of AAV injection, the visual field coverage shown in Figure 1B is most likely an overestimate. The anisotropy was observed in anterogradely (mean aspect ratio ± SEM: 1.4 ± 0.8, *N* = 4) and retrogradely (mean aspect ratio ± SEM: 1.3 ± 0.33, *N* = 4) labeled tangential projections. The anisotropies of injection sites were smaller (anteroAAV 1.15 ± 0.12, retroAAV 1.12 ± 0.1), suggesting that anisotropic tracer uptake played a minor role in the spatial distribution of tangential projections.

### Modular specificity of projection neurons and their apical dendrites

**Figure 3:**
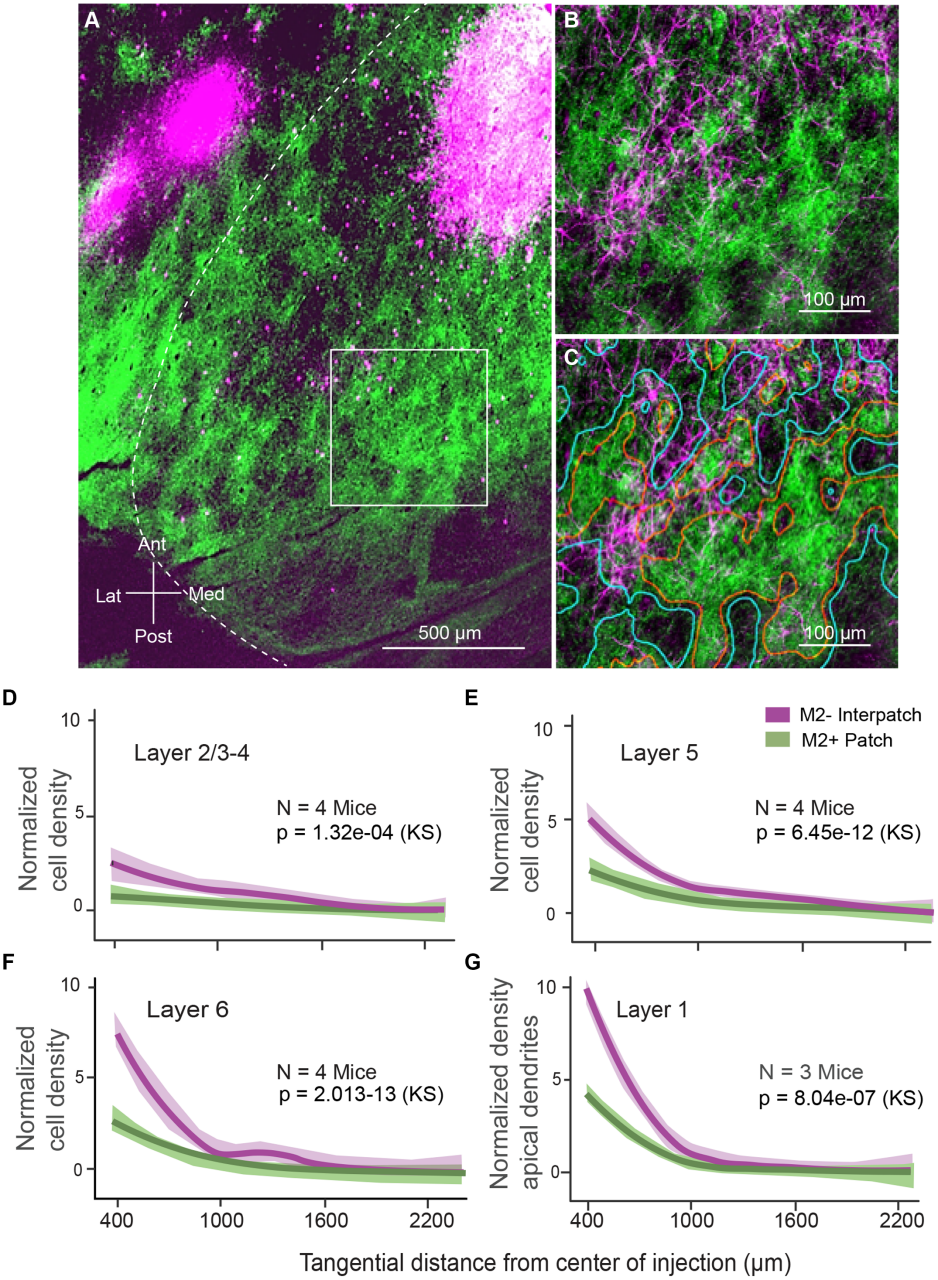
Tangential sections through visual cortex of Ai9 mouse. **(A)** Retrogradely labeled horizontal connections in 2/3 of primary visual cortex (V1) produced by injection of AAV2.Retro-CAG.Cre into V1. Green patches represent axonal projections to L1, labeled by injections of AAV2/1-hSyn-EGFP.WPRE.bGHe into the dLGN. Geniculocortical projections to L1 are known to terminate in M2+ patches (Ji et al., 2015; D’Souza et al., 2019). The image was assembled by aligning serial sections across L1 and L2/3, using blood vessels as landmarks. The size of the injection appears larger than the effective zone of uptake, due to overexposure of the image, required for revealing the cell bodies of local projection neurons. Outside of the intensely fluorescent injection core, distinct retrogradely labeled neurons (magenta) are distributed throughout V1. The distribution is anisotropic: more extensive along elevation than azimuth (for map see Figure 1B). In the surrounding areas, labeled cell bodies are clustered into uniform patches in areas LM (lateromedial) and AL (anterolateral). Notice that despite the large injection in V1, most local projection neurons are found in M2− interpatches. **(B)** Retrogradely labeled dendrites of local horizontally projecting neurons in L1 of V1. Higher magnification image of boxed region shown in **(A)**. Green patches are anterogradely AAV labeled inputs from the dLGN. Most dendritic profiles branch in M2− interpatches. **(C)** Same images as in **(B)**. M2+ patches are outlined in red. M2− interpatches are surrounded by blue lines. **(D–F)** Normalized density of a total of 4,035 retrogradely labeled horizontally projecting neurons. L2/3 **(D)**, L5 **(E)**, and L6 **(F)** of V1, plotted as a function of distance from the core of the injection. The central 600 μm-wide core is discounted due to the high intensity of fluorescence. **(G)** Normalized density of retrogradely labeled apical dendrites of horizontally projecting neurons in V1, showing that apical dendrites are significantly clustered in M2− interpatches. Shading: SEM. KS, Kolmogorov–Smirnov test.

We injected AAV2.Retro.CAG.Cre into V1 of Ai9 (B6,Cg-Gt[ROSA]26Sor^tm9(CAG-tdTomato)Hze^/J) mice (*N* = 4) to retrogradely label locally projecting neurons. Limiting injections to the small diameter of M2+ and M2− modules was challenging. Similar to anterograde AAV, the intense fluorescence at the injection site made it impossible to assign with confidence injections to M2+ patches or M2− interpatches. Larger 300–400 μm-wide injections that spanned multiple M2+ and M2− modules were more successful. Most of the injections (*N* = 4) were placed in the binocular visual field, 20°–40° elevation and 30°–50° azimuth. In each case this resulted in hundreds of retrogradely labeled neurons distributed outside of the injection core (Figure 3A). The labeled cell bodies were distributed widely across an elliptical region, whose long axis mapped elevation and the short axis represented azimuth. Individual neurons were well-labeled, so that not only cell bodies but the entire dendritic arbor was conspicuous and could be plotted across L1-6 (Figures 3B,C; Supplementary Figure S4). Tracing the spatial pattern in L1 of labeled apical dendrites of projection neurons whose cell bodies laid in the layers below was critical for assessing the overlap of these putative postsynaptic targets with anterogradely labeled horizontal axonal projections (Figures 1A,B).

To determine whether cells and apical dendrites were distributed preferentially in M2+ or M2− compartments we anterogradely traced geniculocortical connections, stained sections with an antibody against M2, and partitioned V1 into M2+ and M2− domains, as outlined above. We found that the density of labeled cells in L2/3-4 underneath M2− interpatches was significantly (*p* = 1.32e-02, KS) higher than below M2+ patches (Figure 3D). This indicates that cells bodies formed clusters (Supplementary Figure S4), and hints that M2− interpatch pattern in L1 constrains the spatial distribution of local projection neurons. In L5 and 6 the density of retrogradely labeled cells underneath M2− interpatches was not only higher, but differed significantly from M2+ patches (*p* = 6.45e − 2, KS, *N* = 4 mice, 3 ROIs/mouse; Figures 3E,F). The alignment of cells across L2-6 suggests that local projection neurons are clustered in a columnar architecture (Figures 3E,F).

Retrograde labeling was strong enough to completely label apical dendrites (Supplementary Figure S4), which were readily identified as thin spine-covered branches. The differential distribution of apical dendrites in L1 was even more striking than the clustering of cell bodies in L2-6. Apical dendrites in M2− interpatches were significantly (*p* = 8.04–07, KS, *N* = 4 mice, 3 ROIs/mouse) denser than in M2+ patches (Figure 3G). This indicates that dendrites of local projection neurons overlap with horizontally-projecting axons of V1 neurons, and form looped overlapping interconnections between pyramidal cells representing distant retinotopic locations. Moreover, the results suggest that long-range horizontal axonal connections are preferentially targeted in like-to-like fashion to apical dendrites of the M2− interpatch subnetwork. Thus, what matters for the horizontal network in L1, is that axo-dendritic synaptic contacts are made in M2− interpatches. By comparison the clustering of projection neurons is coarser, emphasizing that the precise location from which an action potential is fired is less important.

## Discussion

Using anterograde and retrograde AAV tracing of local V1 pathways, combined with tracing of dLGN input and M2 immunolabeling to distinguish M2+ patches and M2− interpatches in L1, we have found that horizontal connections preferentially target M2− interpatches in L1. Horizontal axonal connections in L2-6 were weaker and not clustered (Figures 2A–D; Supplementary Figure S3). Unexpectedly, we found clustered connections after large AAV injections involving L1-6, and extending tangentially across multiple M2+ patch and M2− interpatch domains. At first glance, such injections lack the resolution necessary for determining the laminar and modular origin of projections. Despite this, we found that the cell bodies across L2-6 were somewhat clustered underneath M2− interpatches in L1, hinting at a columnar architecture (Figures 2D–F). Unlike the weakly significant patchiness of cell bodies, the projection neurons’ apical dendrites were strongly clustered in M2− interpatches of L1 (Figure 3; Supplementary Figure S4). These labeling patterns indicate that while horizontal L2-6 axons may make contacts with basal dendrites in all layers, and patch and interpatch compartments, inputs to L1 are preferentially targeted to projection neurons with apical dendrites in M2− interpatches. This connectivity pattern indicates that contextual communications between widely separated parts of the visual map occur selectively between M2− interpatch neurons, and that connections are reciprocal and topologically like-to-like. It is worth mentioning that the connectivity pattern we have found applies to the central binocular visual field, and may differ from networks that represent the periphery of the visual map (Saleem and Busse, 2023). Nevertheless, the key feature that emerges is that local long-range synaptic interactions are specifically targeted to apical dendrites of M2− cells. Thus, it is likely that this L1 pathway conveys contextual influences from the RF surround, which modulates the output of M2− neurons.

### Patchy horizontal network for contextual interactions

Patchy horizontal intra-areal projections are well-established networks of V1 in monkey, cat, and tree shrew (Rockland and Lund, 1982; Rockland and Lund, 1983; Martin and Whitteridge, 1984; Blasdel et al., 1985; Gilbert and Wiesel, 1989; Bosking et al., 1997). Such networks are thought to play a role in the integration of locally encoded stimulus features into a global multidimensional representations of space (Martin et al., 2014; Chavane et al., 2022). The main focus of these studies was on patchy interconnections between functional columns, domains that are “not created by the topographic map, but emerge from it” (da Costa and Martin, 2010). Little attention was payed to horizontal projections to L1, the cell-sparse layer, which contains apical dendrites of cells in the layers below and receives feedback projections from higher cortical areas (D’Souza et al., 2022; Burkhalter et al., 2023). Feedback inputs have been shown to gate the effects of horizontal connections, augment contour signals in postsynaptic neurons, and suppress the background noise, to facilitate image segmentation (Liang et al., 2017). The cellular mechanism by which this may occur is by coupling of synchronous inputs arriving at basal dendrites in the middle layers and apical dendrites in L1 (Larkum, 2013).

We have found in mouse V1 that horizonal connections in L1 extend hundreds of microns beyond the ~400 μm-wide injection core and terminate preferentially in M2− interpatches (Figures 1A,B). The core contains approximately 30,000 neurons (Hercolano-Houzel et al., 2013) underneath a ~260 × 360 μm tangential region that includes a total of 4–8 M2+ patches and M2− interpatches, depending on topographic location (Ji et al., 2015). Together these cells encode the point image, which represents the RF center (Ji et al., 2015). Tangential connections which are longer than 400 μm, therefore, connect distant points of the visual map. Because the connections are reciprocal (Figure 2A) they must provide input from the RF surround.

Unexpectedly, we found that these contextual interconnections strongly prefer M2− interpatches. They do so across a region of the visual field, which is elongated along elevation and is shorter along the azimuthal axis (Figure 2B). This axis-dependence captures a feature first described in tree shrew, where the axis in cortex corresponds to the preferred orientations between laterally displaced, topographically distant neurons (Bosking et al., 1997). It has been proposed that a similar organization exists on mouse V1 (Iacaruso et al., 2017). If so, unlike the orientation-dependent long axis in tree shrew (Bosking et al., 1997), the major axis in mice remains constant, and represents a reciprocal horizontal network which is patchy and specifically linked to M2− interpatches. Such a network could be called like-to-like. However, studies in cat V1 have shown that lateral connections are more heterogenous and not only link preferred orientations, but connect cells tuned to similar spatial frequencies, temporal frequencies and directions of motion (Martin et al., 2014; Chavane et al., 2022). Based on our earlier findings, all of these visual properties are represented at the injection site, with a higher density of orientation selective, high spatial frequency tuned cells in M2+ patches, and a greater density of direction, speed and motion coherence tuned cells in M2− interpatches (Ji et al., 2015). If our AAV injections were equally effective in transfecting M2+ and M2− cells and labeling all of their connections to L1, as our findings indicate (Figures 1C,D), the absence of long patchy M2+ connections suggest that these connections are shorter than the patchy M2− connections. This connectivity pattern differs form that found in L2/3 of monkey V1, where horizontal connections are roughly equal in length in all direction and show preferences for either cytochrome-rich blobs or cytochrome-poor interblobs (Malach et al., 1993; Stettler et al., 2002). The L1 connections in mouse V1 presumably originate from different cell types in L2/3, 5 and 6 (Burkhalter, 1989; Han et al., 2018) in M2+ patches or M2− interpatches, whose local projections are either short or extend beyond the point image, respectively. Support for such an organization comes from tracing experiments in rat V1, which showed that L2/3 cells that project to lateral higher visual areas have short tangential connections in V1, while cells which project to medial visual areas have long local connections (Burkhalter and Charles, 1990). Notably, in mouse projections to the medial area PM originate from M2− interpatches, which further strengthens the proposal that long tangential connections are selective for M2− interpatches.

M2− interpatch cells stand out by the tuning of the RF center to random dot kinematograms (Ji et al., 2015). While this sensitivity is necessary for processing of optic flow, extracting the flow’s direction and make it orientation independent requires integration across large parts of the visual field (Prince and Born, 2009). This may be achieved by long horizontal connections, particularly those along the vertical axis, which represents the dominant flow pattern during locomotion (Saleem, 2020). Horizontal connections in mouse V1 may favor stimulus directions perpendicular to the orientation of the contour stimulating the RF, and exhibit features similar to intracortical feedback connections (Marques et al., 2018).

### Overlap of patchy horizontal V1 connections with other networks

In monkey horizontal V1 connections interact with feedback connections from V4 (Barone et al., 2000; Gilbert and Li, 2013; Liang et al., 2017). While the connections terminate in L1 of V1, it is not known whether they are patchy (Rockland et al., 1994). That feedback connections to L1 can be patchy has been shown in the pathway from V2 to V1, where inputs from thin stripes are aligned with blobs in L2/3, whereas inputs from pale and thick stripes line up with interblobs (Federer et al., 2021). Although feedback projections from V2 overlap with horizontally projecting neurons in V1, the synaptic interactions are indirect (Sui et al., 2021).

Patchy feedback projections from higher visual cortical areas have also been found in L1 of mouse V1 (Ji et al., 2015). In mice, feedback inputs from the ventral stream area LM (Wang et al., 2012), and from a subset of lateral dorsal stream areas, AL (anterolateral) and RL (rostrolateral; Wang et al., 2012), terminate in M2+ patches (D’Souza et al., 2019, 2021). In contrast, feedback input to V1 from the medial dorsal area PM (posteromedial) and the secondary motor cortex (MOs) terminate in M2− interpatches, where they may contact the apical dendrites of the reciprocal feedforward projecting neurons in layers 2–6 (D’Souza et al., 2019). Notably, these feedback inputs together with inputs from the LP thalamus (i.e., pulvinar) overlap with inputs from the horizonal network in V1, which may play a role in emergent retina-independent direction selectivity (Rasmussen et al., 2020), suggesting that interactions between these networks are specific to M2− interpatches. LP inputs have been shown to provide visual directional object motion signals (Roth et al., 2016) and non-visual motion signals from saccades to V1 (Miura and Scanziani, 2022). Thus, interactions between converging signals from the LP, horizontal and feedback connections to M2− interpatches may play a role in the discriminating externally-generated from self-generated visual inputs.

## Data availability statement

The raw data supporting the conclusions of this article will be made available by the authors, without undue reservation.

## Ethics statement

The animal study was approved by Institutional Animal Care and Use Committee Washington University in St. Louis. The study was conducted in accordance with the local legislation and institutional requirements.

## Author contributions

AB: Conceptualization, Funding acquisition, Methodology, Project administration, Resources, Supervision, Writing – original draft, Writing – review & editing. WJ: Data curation, Investigation, Methodology, Validation, Visualization, Writing – review & editing. AM: Data curation, Investigation, Methodology, Software, Writing – review & editing. RD’S: Data curation, Formal analysis, Investigation, Methodology, Validation, Writing – review & editing.

## Supplementary material

SUPPLEMENTARY FIGURE S1 Tangential section through V1 of C57BL/6J mouse. **(A)** Non-uniform, pattern of geniculocortical fibers in L1 (i.e. proxy for M2+ patches, red contours), labeled by tracing by injections of AAV2/1-hSyn-EGFP.WPRE.bGHe into the dLGN. **(B)** Same image as in (A), showing anterogradely labeled tangential axonal connections near the injection site of AAV2/1-hSyn-tdT.WPRE.bGHe. At the core (solid white line) of the injection labelling is uniform and shows no distinct structural features. The core is surrounded by a halo (dashed white line), which shows labeled fibers that freely cross the compartments innervated or not innervated by geniculocortical afferents. Outside of the halo, fibers are organized into clusters, which preferentially innervate interpatch domains that lack geniculocortical input (blue contours). **(C)** Merge of A and B, showing that tangential projections outside of the halo have s strong preference for M2- interpatches.

SUPPLEMENTARY FIGURE S2 Identification of layers in flatmounted visual cortex. **(A)** Coronal section through occipital cortex of Pvalb-Cre (B6, 129Ps-Pvalbtm1(cre)Arbr/J) crossed with Ai9 reporter mouse in which tdTomato is expressed in cell bodies and processed of parvalbumin neurons, which are enriched in L2/3 and 4. The section is stained with an antibody against the transcription factor, Ctip2, which preferentially labels neurons in L5 and 6. The laminar PV and Ctip2 expression patterns are then converted into numbers of 40 μm sections cut in the tangential plane. Tangential sections, aligned to the pial surface, are used to assign layers. **(B–C)** Tangential 40 μm sections through left cerebral cortex stained for Ctip2. Notice that in V1 Ctip2 expression is absent in section 4 (B), but is present in the deeper sections 8 (C) and 12 (D). **(B’–D’)** Merge of B–D. Abbreviations: ACA (anterior cingulate area), AI (anterior insular cortex), AUD (auditory cortex), CP (caudate putamen), Entl (lateral entorhinal cortex), Entm (medial entorhinal cortex), Hip (hippocampus), OB (olfactory bulb), ORB (orbitofrontal area), OT (olfactory tubercle), Pir (piriform cortex), PL (prelimbic area), PM (posteromedial area), RSP (retrosplenial cortex), SSp (primary somatosensory cortex), SSs (secondary somatosensory cortex), V1 (primary visual cortex).

SUPPLEMENTARY FIGURE S3 Horizonal connections in tangential section through L2/3 of V1. **(A)** Axonal projections labeled by anterograde tracing with AAV2/1-hSyn-EGFP.WPRE.bGHe showing a non-clustered distribution relative to patches (surrounded by red lines) and interpatches (surrounded by blue lines). **(B)** M2+ immunolabeled patches in same image as shown in A. **(C)** Overlay of A and B, showing that tangential projections in L2/3 have no preference for patches or interpatches.

SUPPLEMENTARY FIGURE S4 Horizontal section that contains the bottom of L1 and the top of L2/3. **(A)** Dendrites in L1 (arrow heads) of retrogradely labeled L2/3 cells (arrows), after an injection of AAV2.Retro-CAG.Cre into V1 of an Ai9 tdT reporter mouse. **(B)** Immunolabeling with an antibody against M2, showing M2+ patches (outlined by red contours) and M2− interpatches (outlined by blue contours). **(C)** Overlay of A and B, showing that retrogradely labeled dendrites in L1 and cells in L2/3 are preferentially contained in M2− interpatches.
