# Supplementary figures and images for: Modular horizontal network within mouse primary visual cortex
Source: Front Neuroanat. 2024 Apr 8;18:1364675. doi: 10.3389/fnana.2024.1364675 (PMC11033472; doi:10.3389/fnana.2024.1364675)

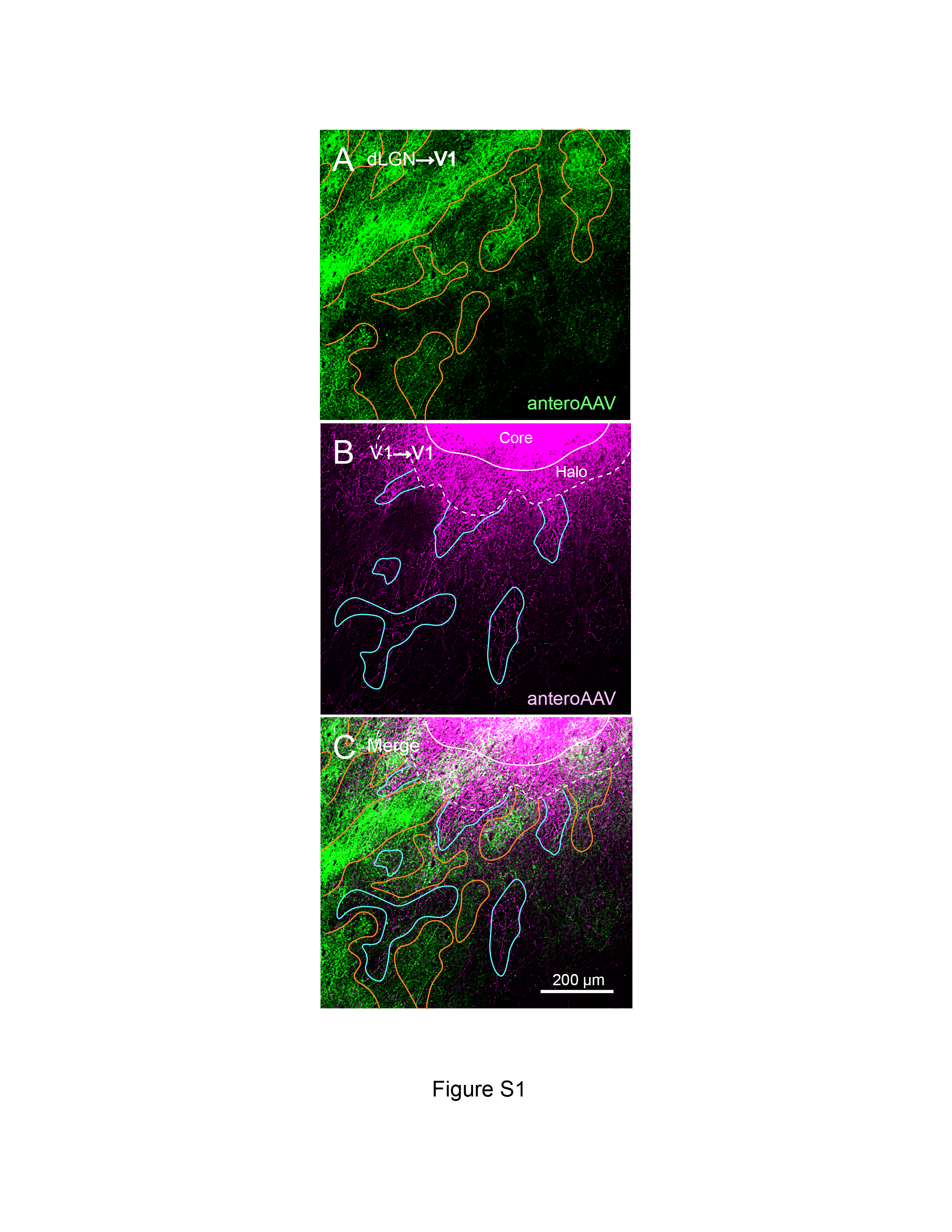

Supplement: SUPPLEMENTARY FIGURE S1 — Tangential section through V1 of C57BL/6J mouse. (A) Non-uniform, pattern of geniculocortical fibers in L1 (i.e. proxy for M2+ patches, red contours), labeled by tracing by injections of AAV2/1-hSyn-EGFP.WPRE.bGHe into the dLGN. (B) Same image as in (A), showing anterogradely labeled tangential axonal connections near the injection site of AAV2/1-hSyn-tdT.WPRE.bGHe. At the core (solid white line) of the injection labelling is uniform and shows no distinct structural features. The core is surrounded by a halo (dashed white line), which shows labeled fibers that freely cross the compartments innervated or not innervated by geniculocortical afferents. Outside of the halo, fibers are organized into clusters, which preferentially innervate interpatch domains that lack geniculocortical input (blue contours). (C) Merge of A and B, showing that tangential projections outside of the halo have s strong preference for M2- interpatches. [file Image_1.tif]

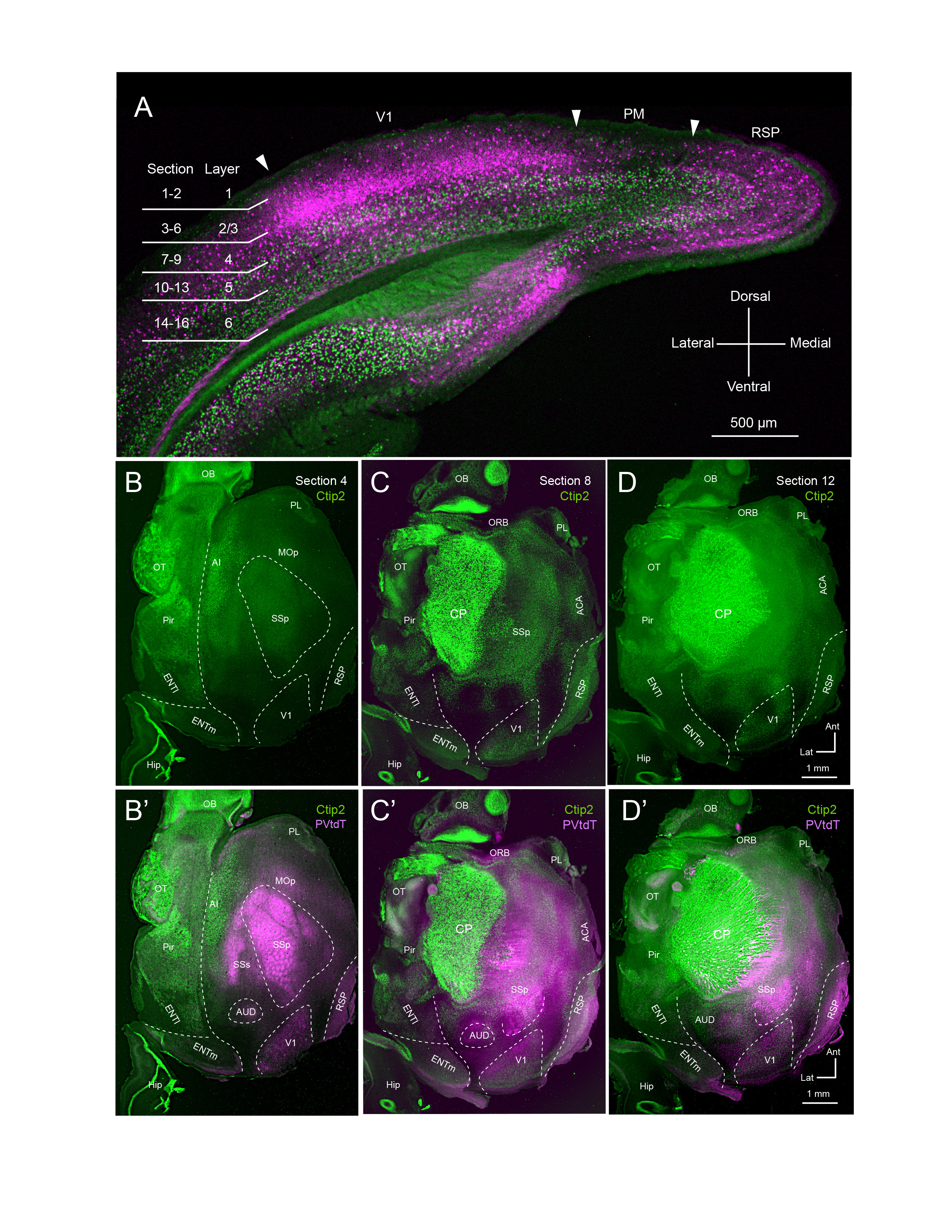

Supplement: SUPPLEMENTARY FIGURE S2 — Identification of layers in flatmounted visual cortex. (A) Coronal section through occipital cortex of Pvalb-Cre (B6, 129Ps-Pvalbtm1(cre)Arbr/J) crossed with Ai9 reporter mouse in which tdTomato is expressed in cell bodies and processed of parvalbumin neurons, which are enriched in L2/3 and 4. The section is stained with an antibody against the transcription factor, Ctip2, which preferentially labels neurons in L5 and 6. The laminar PV and Ctip2 expression patterns are then converted into numbers of 40 μm sections cut in the tangential plane. Tangential sections, aligned to the pial surface, are used to assign layers. (B–C) Tangential 40 μm sections through left cerebral cortex stained for Ctip2. Notice that in V1 Ctip2 expression is absent in section 4 (B), but is present in the deeper sections 8 (C) and 12 (D). (B’–D’) Merge of B–D. Abbreviations: ACA (anterior cingulate area), AI (anterior insular cortex), AUD (auditory cortex), CP (caudate putamen), Entl (lateral entorhinal cortex), Entm (medial entorhinal cortex), Hip (hippocampus), OB (olfactory bulb), ORB (orbitofrontal area), OT (olfactory tubercle), Pir (piriform cortex), PL (prelimbic area), PM (posteromedial area), RSP (retrosplenial cortex), SSp (primary somatosensory cortex), SSs (secondary somatosensory cortex), V1 (primary visual cortex). [file Image_2.jpg]

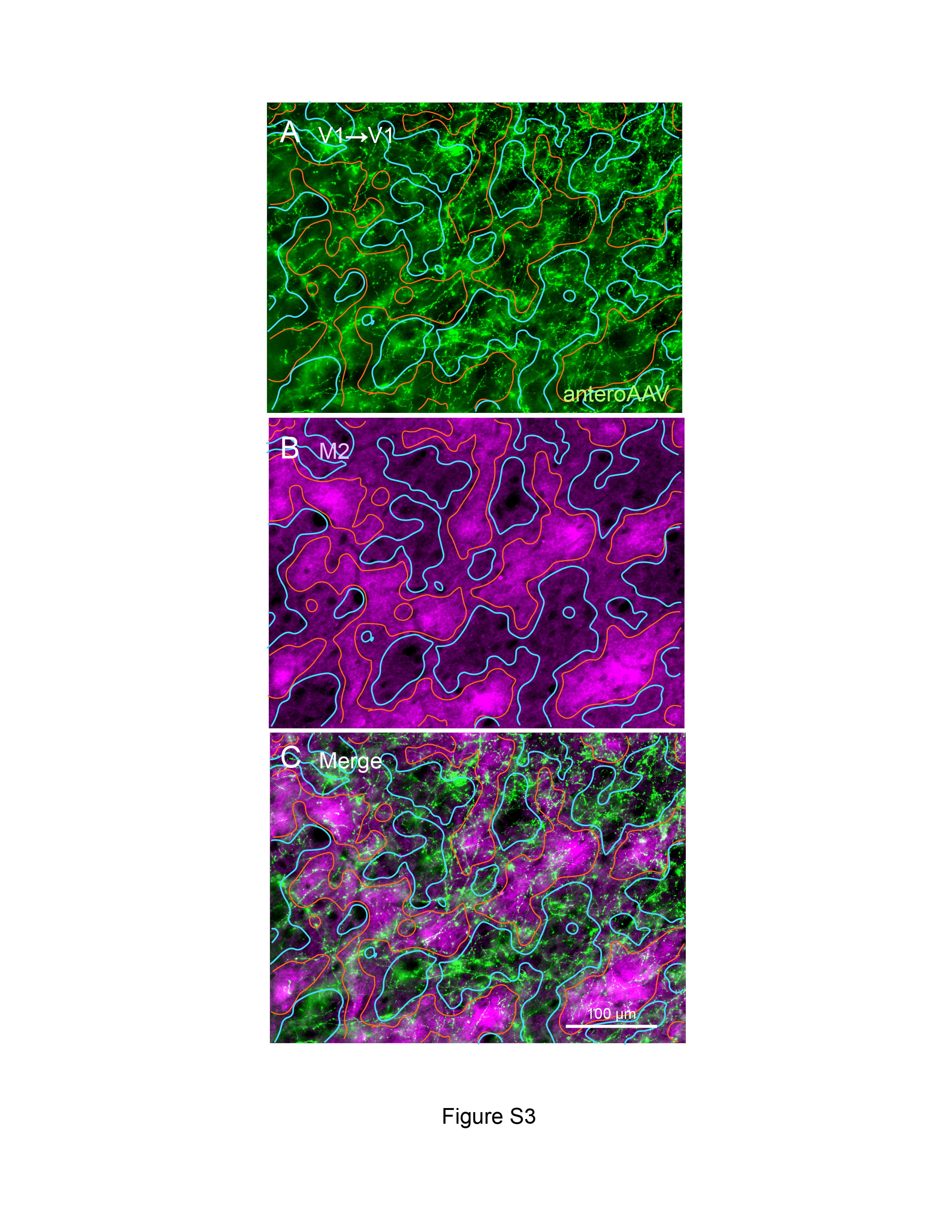

Supplement: SUPPLEMENTARY FIGURE S3 — Horizonal connections in tangential section through L2/3 of V1. (A) Axonal projections labeled by anterograde tracing with AAV2/1-hSyn-EGFP.WPRE.bGHe showing a non-clustered distribution relative to patches (surrounded by red lines) and interpatches (surrounded by blue lines). (B) M2+ immunolabeled patches in same image as shown in A. (C) Overlay of A and B, showing that tangential projections in L2/3 have no preference for patches or interpatches. [file Image_3.tif]

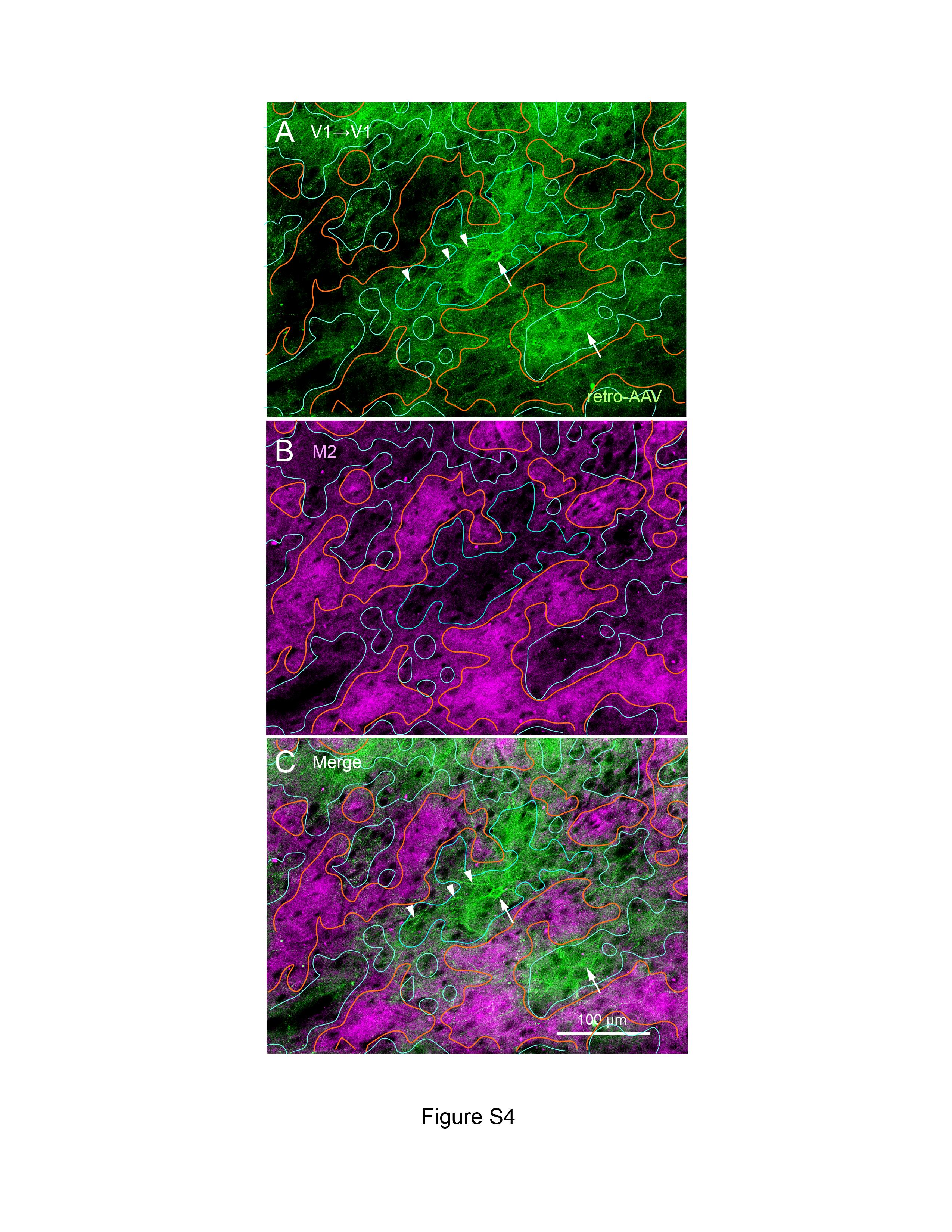

Supplement: SUPPLEMENTARY FIGURE S4 — Horizontal section that contains the bottom of L1 and the top of L2/3. (A) Dendrites in L1 (arrow heads) of retrogradely labeled L2/3 cells (arrows), after an injection of AAV2.Retro-CAG.Cre into V1 of an Ai9 tdT reporter mouse. (B) Immunolabeling with an antibody against M2, showing M2+ patches (outlined by red contours) and M2− interpatches (outlined by blue contours). (C) Overlay of A and B, showing that retrogradely labeled dendrites in L1 and cells in L2/3 are preferentially contained in M2− interpatches. [file Image_4.tif]
